# Happiness and Social Exclusion of Indigenous Peoples in Taiwan - A Social Sustainability Perspective

**DOI:** 10.1371/journal.pone.0118305

**Published:** 2015-02-19

**Authors:** Jiun-Hao Wang

**Affiliations:** Department of Bio-industry Communication and Development, National Taiwan University, Taipei, Taiwan, Republic of China; Bournemouth University, UNITED KINGDOM

## Abstract

**Introduction:**

Happiness and social inclusion are important indicators of social sustainability, as recommended in the Sustainable Development Goals; however, little is known about the social sustainable development of ethnic minorities. To fill this knowledge gap, special attention is paid to understanding the issues of social exclusion and happiness in relation to the indigenous peoples in Taiwan.

**Methods:**

Data used were drawn from a nationwide representativeness survey of the Taiwanese Indigenous People in 2007; it included 2,200 respondents. This study employed binary logistic regression to examine the effects of different domains of social exclusion on the likelihood of perceiving happiness; other exogenous factors, were controlled.

**Results:**

The results show that among the respondents, mountain indigenous peoples, females, the elderly and those who are healthier, wealthier, highly educated, possessing western beliefs, and are more likely to be happy, compared to their counterparts. As expected, the results reveal that the likelihood of being happy is higher for those who have received medical benefits, as well as those persons without housing problems or financial difficulties, compared to their excluded counterparts. However, no significant association is found between happiness and some social exclusion domains, such as child and youth benefits, and unemployment benefits.

**Conclusions:**

The disengagement of the indigenous peoples in mainstream society, with respect to the accessibility of welfare provisions, is a crucial element in regard to social exclusion and happiness. Several policy implications for the social sustainability of indigenous peoples can be inferred from these findings. For example, providing a mobile clinical tour, on-site health counseling, or homecare service can contribute to the removal of institutional and geographic barriers to medical welfare provisions for the mountain indigenes. Moreover, the government may devote more welfare resources to assist indigenous families and tribal communities to develop their own social safety net, instead of the individual-oriented welfare provisions.

## Introduction

Given the expressed concerns for social sustainability, the promotion of Sustainable Development Goals (SDGs) has achieved a broad consensus in the Rio+20 Earth Summit [1]. The United Nations (UN) conference on sustainable development reached the conclusion that Bhutan’s Gross National Happiness (GNH) initiative had become a relevant pillar for social development policy [2]. In addition to happiness, achieving social inclusion for all is also an important indicator of social sustainability; this perspective will be recommended by the forthcoming post-2015 development agenda of the UN [1, 3].

Although a considerable body of literature has examined the determinants of happiness of diverse groups in Taiwan, relatively little is known about the ethnic minorities’ subjective wellbeing [4–7]. To fill this knowledge gap, special attention is paid herein to understanding the issues of happiness and institutional exclusion in regard to the social sustainable development of the indigenous peoples in Taiwan.

Societal progress has traditionally been measured in terms of general economic growth or Gross Domestic Product (GDP) per capita. Previous studies have addressed the negative impacts of the economic growth approach on sustainable development, including social inequalities, poverty, unhappiness and environmental degradation [8]. In contrast to the economic growth model, the Bhutanese GNH paradigm moves beyond GDP towards sustainable long-term social progress. The social progress approach of the GNH aims at enhancing spiritual wellbeing, environmental improvement and social equity, rather than just focusing on material living conditions [2, 9]. Similarly, the United Nations Statistics Office should support countries to look beyond gross domestic product and propose the SDGs as a new developmental indicator to measure progress in human wellbeing. Given the spatial and environmental constraints inherent in the remote indigenous communities, any development policy which overemphasizes economic performance, capital accumulation and productivity improvements will be problematic. Current trends within the indigenous development policy and practices, particularly development experiences of ethnic groups in Bhutan, the Maori in New Zealand and the Menominee in the USA, suggest that enhancing happiness and social inclusion is an appropriate approach for the indigenous peoples in remote areas [10]. Consequently, it is encouraging to observe the social progress made with regard to increasing indigenous peoples’ subjective wellbeing and ensuring their access right to social welfare programs on a sustainable basis.

With regard to the current situation of the indigenous peoples in Taiwan, there are, in total, 530,000 indigenous peoples, accounting for 2.3% of the overall population; this ratio is similar to that of Australia or Canada [11, 12]. Although there are 14 distinctive tribes officially recognized by the Council of Indigenous Peoples (CIP) in Taiwan, the indigenous groups are generally divided into plains and mountain categories: Pingpu (Plains tribes) and Gaoshan (Mountain tribes), based on their geographical distribution. The mountain aborigines account for 53% of all indigenous peoples; a quarter of them live in remote mountain areas [13]. Due to the natural constraints of living in traditional territories, namely geographic remoteness, spatial isolation and high vulnerability to disasters, the mountain indigenous peoples are not only national minorities, but also one of the most disadvantaged groups in Taiwan, in socio-economic terms.

In addition, the life expectancy at birth and the unemployment rate for indigenous peoples were 70.1 years and 8.85%, respectively, in 2009. Compared to 79.0 years and 6.04% for other Taiwanese, i.e. the indigenous peoples have 9 years less in life expectancy at birth and 2.81% more in their unemployment rate, respectively, in data for 2009 [14]. Furthermore, the socio-economic status of indigenous peoples is characterized by poverty, substance abuse, alcoholism, relative lower socio-economic status, political marginalization and suffering discrimination in Taiwanese society [13]. For example, the remote indigenous mountain communities did not evenly share the benefits of economic growth in Taiwan over the past decades. Second, in their everyday life, they face challenging obstacles in regard to sustainable development, especially in their lack of infrastructure, poor access to welfare provisions, and insufficient education and job opportunities; these factors resulted in many social barriers imposed on indigenous peoples in mountain areas. Consequently, it is evident that the social development issues and policies of indigenous peoples different from those of mainstream society.

In order to narrow the socio-economic gap between the indigenous peoples and the general population, the Taiwanese government has launched several specific social welfare and benefit programs for the indigenous peoples. The cash payments to indigenous peoples include old-age indigenes' welfare living allowance (for those aged 55–64 years), aid for emergencies confronting indigenes, scholarships for indigenous education development and tuition subsidies. Moreover, social benefits for indigenous peoples include: child care and teaching subsidies for indigenous children, school lodging and meal allowance for indigenous students, subsidies for employment and career plans, loans, National Health Insurance subsidies, medical allowance (i.e. transportation subsidies for those seeking medical advice), interest subsidies for building and repairing houses or for house rent and community reconstruction [14].

This paper focuses on the social dimensions of the indigenous people’s development. Special attention is paid to understanding their social exclusion and happiness or unhappiness. The objective of this study was twofold. First, a nationwide representativeness survey was used to investigate the sample distributions of the different domains of social exclusion and the happiness status of the indigenous peoples. Secondly, this study distinguished the extent to which different social exclusion status and socio-demographic characteristics are associated with the likelihood of perceiving happiness among the indigenous peoples. The remainder of this article is organized as follows. The next section briefly introduces the data used in this study. The following section then presents an empirical model and the results obtained. The final section concludes this article with a brief summary and a discussion of policy implications.

## Material and Methods

### Data

Data used in this study were drawn from the “Social Change and Policy of Taiwanese Indigenous Peoples Survey” (TIPS) in 2007, conducted by the Institute of Ethnology in Academia Sinica [15]. The survey targeted indigenous adults and followed a systematic sampling scheme, which took the population distribution of indigenous peoples into account, according to the official household registration records of the Ministry of the Interior in Taiwan. To ensure the nationwide representativeness, a two-stage stratified probability proportional to size (PPS) sampling design was employed in the survey. In addition, trained interviewers conducted face-to-face interviews at participants’ homes to ensure a high quality of data collection; the respondents included 2,299 indigenous peoples aged 18–65. Data used in this study combined information derived from 2,057 respondents of the original survey and 242 observations of an additional survey in eastern Taiwan [15].

After deleting those with missing values in terms of key variables, such as social exclusion related items, 2,200 respondents remained for use in our analysis. The TIPS data include self-reported happiness and detailed social exclusion information, such as government provided in-kind benefits and cash payments due to economic difficulties and special social needs. In addition to public welfare provisions, several variables representing the socio-demographic characteristics of the respondents were specified, including gender, age, education, income, religion, health status and residential area.

### Measurement

#### Measure of happiness

Given that the attainment of happiness plays a crucial role in achieving the goals of social sustainable development [2], the concept of happiness used in this analysis concerns both overall judgments about the quality of life and life-as-a-whole. Happiness represents the subjective degree to which persons judge their entire life quality favorably, also known as overall happiness [16]. In the TIPS questionnaire, happiness was measured by the question: ‘Taking all things together, would you say you are, on the whole: Very happy, quite happy, fair, unhappy, or not at all happy?’ (A 5-point Likert-type scale was coded from 1 = very happy, to 5 = not at all happy). Due to the low percentage of responses in the “not at all happy” category (3.0%), this study employed a dummy variable indicating whether the interviewees evaluate their overall perception of their life-as-a-whole quality. If they chose unhappy or not at all happy, it would be recorded in the unhappy category (= 0); others were recorded as happy (= 1).

#### Measures of social exclusion

The independent variable of particular interest in this study is social exclusion. Sen (2000) indicated that social exclusion refers to the processes in which marginalized groups are systematically excluded from full participation in the society in which they live, especially being blocked from rights, opportunities and resources that are normally available to members of that society [17]. From the perspective of social sustainability, actions to combat social exclusion concentrate on welfare provision, including: cash transfer, employment, healthcare, housing and other social services delivery. Thus, this study hypothesizes that the different excluded situations from social welfare benefits are associated with the happiness of the indigenous peoples.

Based on an institutional perspective, social exclusion can be recognized as the relationships with the main welfare agents, and the accessibility of welfare provisions, together with its gaps and failings [18]. For example, although the National Health Insurance program provides a universal right to healthcare for all Taiwanese, the indigenous peoples are often excluded from participation in healthcare programs or are unable to access medical benefits due to insufficient physicians and clinics in remote areas. Considering the institutional basis and dimensions of social exclusion [18, 19], this study defines social exclusion as the lack of accessibility of different welfare provisions.

Due to the social exclusion’s multidimensional character, it was measured by several dummy indicators; its welfare provisions were investigated in the TIPS dataset, including: health and medical care, housing benefits, child and youth benefits, senior benefits, as well as unemployment and financial subsidies. Each indicator of social exclusion was divided into three categories: no need to apply for, not excluded and excluded from the specific welfare provision. Taking the excluded category (as reference group) for example, the dummy variable indicates whether the respondent has a need for specific welfare provision but cannot access the corresponding social services or benefits from the public sectors (= 1 if not received). Detailed operational definitions of the social exclusion variable are presented in Table 1.

**Table 1:** Definitions and summary statistics of regression variables (N = 2,200).

Variable	Definition and measurement	%
**Happiness**	If the respondent self-reports as happy (= 1)	0.87
**Social exclusion domains**		
*Medical benefits*		
no need	If the respondent has no need for any medical benefit (= 1), e.g., medical care, health check, health counseling, National Health Insurance subsidies for indigenous peoples, etc.	0.23
not excluded	If the respondent needs and receives medical benefits (= 1)	0.47
excluded	If the respondent could not receive needed medical benefits (= 1)	0.30
*Housing benefits*		
no need	If the respondent has no need for housing benefits (= 1), e.g.,house repair subsidy, rent subsidy, housing concessionary loan, etc.	0.24
not excluded	If the respondent needs and receives housing benefits (= 1)	0.23
excluded	If the respondent could not receive needed housing benefits (= 1)	0.53
*Child and youth benefits*		
no need	If the respondent has no need for child & youth benefits (= 1), e.g., child care allowance, free lunch for low-income students, etc.	0.31
not excluded	If the respondent needs and receives child & youth benefits (= 1)	0.28
excluded	If the respondent could not receive needed child & youth benefits (= 1)	0.41
*Senior benefits*		
no need	If the respondent has no need for senior benefits (= 1), e.g., elderly home care, meal delivery, old-age welfare living allowance, etc.	0.45
not excluded	If the respondent needs and receives senior benefits (= 1)	0.15
excluded	If the respondent could not receive needed senior benefits (= 1)	0.40
*Unemployment benefits*		
no need	If the respondent has no need for unemployment benefits (= 1), e.g., unemployment subsidy, employment assistance, vocational training etc.	0.62
not excluded	If the respondent needs and receives unemployment benefits (= 1)	0.12
excluded	If the respondent could not receive needed unemployment benefits (= 1)	0.26
*Financial assistances*		
no need	If the respondent has no need for financial assistance (= 1), e.g., aid for emergency for Indigenous Peoples, emergency assistance for livelihood, living support for low-income family, etc.	0.38
not excluded	If the respondent needs and receives financial assistance (= 1)	0.06
excluded	If the respondent could not receive needed financial assistance (= 1)	0.56
**Socio-demographic characteristics**		
*Gender*	If the respondent is male (= 1)	0.44
*Age* (42.33±12.26)	If the respondent’s age is under 55 years (= 1)	0.84
*Educational level*		
Elementary or lower	If graduated from elementary school or uneducated (= 1)	0.27^a^
Junior high school	If graduated from junior high school (= 1)	0.22
Senior high school	If graduated from senior high school (= 1)	0.13
Vocational school	If t graduated from vocational school (= 1)	0.20
College or higher	If graduated from college or higher (= 1)	0.18
*Health status*		
Physical health	If the respondent self-reports as healthy (= 1)	0.45
Mental health problem	If the respondent experienced any mental health problem recently, e.g. anxiety, or mood disorders (yes = 1)	0.77
**Other external factors**		
*Household income*	Average monthly income per household (NT$ 10,000)	4.20±3.30
*Religion*		
Protestant	If the respondent is a Protestant (= 1)	0.41
Catholic	If the respondent is a Catholic (= 1)	0.24
Buddhist or others	If the respondent is a Buddhist or other believer (= 1)	0.35
*Geographic region*		
Remote mountain area	If the respondent lives in remote mountain area (= 1)	0.14
Highlands area	If the respondent lives in highlands area (= 1)	0.39
Plains area	If the respondent lives in plains area (= 1)	0.47

^a^Since each educational category is coded as a dummy variable, the value "0.27" means that 27% of respondents have graduated from elementary school or are uneducated.

#### Measures of control variables

The recent World Happiness Report (2012) summarized the major findings of previous happiness studies and identified two types of determinants of happiness. The personal factors include mental and physical health, family experience, education, gender and age. On the other hand, income, work, community and governance, and religion are some of the important external factors [2]. Since the primary research focus is on examining the relationship between social exclusion and happiness of the indigenous peoples, this study selected several control variables as follows.

The socio-demographic variables include gender (= 1 if male), age (= 1 if aged over 55 years, the cut-off age at 55 is the requirement for applying for the Old-age Indigenes' Welfare Living Allowance in Taiwan) and educational level, categorized into several dummy variables (including elementary or lower, junior high school, senior high school, vocational school, and college or higher). Other health variables were identified as being well, including physical health status (= 1 if in good health), and having mental health problems (= 1 if one of the listed mental disorder symptoms was chosen). Aside from socio-demographic variables, the external factors used in the empirical model are household income, defined as average monthly income (including salary, interest income and government subsidy, in NT$ 10,000 per month), religious beliefs, such as Protestantism, Catholicism, Buddhism, or other religion (= 1 if they chose one).

In addition to the abovementioned relevant external determinants, this study also controls for the residential variable, reflecting the remoteness and lifestyles of the indigenous peoples. All respondents were divided into three geographic regions according to the official documentation of the Council of Indigenous Peoples in Taiwan, namely, the remote mountain areas (refers to high and medium altitude mountain), highland areas (refers to low altitude mountains) and plains areas [20]. Detailed definitions and descriptive statistics of all variables are presented in Table 1.

### Statistical analysis

Due to the happiness being documented as a binary variable (happy = 1; unhappy = 0), this study employs a binary logistic regression model to examine the effects of different domains of social exclusion on the likelihood of perceiving happiness, while controlling for individual socio-demographic characteristics and other exogenous factors, as shown in the following equation:

$$\begin{array}{l} \ell \text{n(}\frac{\text{Probability of happiness}}{\text{Probability of unhappiness}}{\text{)=α + β}}_{\text{1-12}}{\text{ Social Exclusion + β}}_{\text{13-20}}{\text{ Personal factors + β}}_{\text{21-25}}\text{ External factors +ε} \end{array}$$

The statistical analysis contains three steps. Starting with the descriptive statistics of the full sample, the first part presents the happiness, social exclusion, and personal and external characteristics of all the interviewees. Secondly, a group comparison design was used to compare happy and unhappy respondents in terms of their social excluded situations and socio-demographic characteristics. In the final stage, binary logistic regression is estimated to examine the association between social exclusion and exogenous factors, and the likelihood of perceiving happiness. In addition to logistic regression coefficients, the marginal effects are also reported. All the statistical analyses were implemented with the statistical software SAS 9.2 [21].

## Results and Discussion

### Descriptive statistics of the sample characteristics

The sample statistics of the selected variables are presented in Table 1. Among the 2,200 respondents, of which 44% are male, with average age of 42.33 (SD±12.26), nearly half of them graduated from junior high school or below, 41% are Christian, and with average household income NT$ 42,000 (Taiwan Dollars) per month, equivalent to approximately USD 1,448. The self-reported health status of respondents is 45% and 23%, signifying good physical and mental conditions, respectively. It also shows that 53% of the respondents live in mountainous or highland area.

In regard to the social sustainability indicators, it is evident that the ratio of happy to unhappy interviewees is 6.4 (86.5% vs. 13.5%). This result is in accordance with the previous findings of happiness studies in Taiwan [4, 6, 22, 23]. Moreover, the situation of social exclusion varies in different domains of welfare provisions. Financial assistance and housing benefits are the most widely observed exclusion situations in this study. For those who need financial assistance and housing benefits but cannot receive welfare resource accordingly, the figures are 56% and 53%, respectively. In addition, the proportions of other domains of social exclusion, from low to high order are: 26%, 30%, 40% and 41% for unemployment, medical care, seniors, as well as child and youth benefits, respectively.

### Group comparison between happy and unhappy indigenous peoples

A group comparison of happy and unhappy indigenous peoples is reported in Table 2. Based on the Chi-square and t-test results, most of the exogenous factors between happy and unhappy groups were statistically significant (p<.001), except for gender. In general, those who self-reported as being happy (86.5%) were younger, more highly educated, richer, physically and mentally healthier, and with western beliefs, as compared with unhappy counterparts. The regional heterogeneity in perceived happiness is also statistically significant (p<.001).

**Table 2:** Group comparison between happy and unhappy indigenous peoples (N = 2,200).

Explanatory variables	Unhappy (n = 297)	Happy (n = 1,903)	X^2^/t-test
**Social exclusion domains**			
*Medical benefits*			14.82***
no need	0.17	0.24	
not excluded	0.44	0.47	
excluded	0.39	0.29	
*Housing benefits*			25.85***
no need	0.13	0.25	
not excluded	0.22	0.24	
excluded	0.65	0.51	
*Child and youth benefits*			3.78
no need	0.36	0.31	
not excluded	0.27	0.28	
excluded	0.37	0.41	
*Senior benefits*			3.39
no need	0.42	0.45	
not excluded	0.19	0.15	
excluded	0.39	0.40	
*Unemployment benefits*			17.60***
no need	0.51	0.64	
not excluded	0.17	0.11	
excluded	0.32	0.25	
*Financial assistances*			86.86***
no need	0.14	0.42	
not excluded	0.08	0.06	
excluded	0.78	0.52	
**Socio-demographic characteristics**			
*Gender*			1.07
Male	0.47	0.44	
Female	0.53	0.56	
*Age* (42.33±12.26)^a^	46.55±11.12^a^	41.67±12.30^a^	6.43^b^ ***
under 55 years	0.77	0.85	9.31**
over 55 years	0.23	0.15	
*Educational level*			75.08***
Elementary or lower	0.34	0.21	
Junior high school	0.24	0.20	
Senior high school	0.12	0.13	
Vocational school	0.16	0.24	
College or higher	0.14	0.22	
*Health status*			
Physical health status (good)	0.24	0.48	63.65*
Mental health problem (yes)	0.94	0.74	60.12*
**Other external factors**			
*Household income*	2.89±2.79^a^	4.41±3.32^a^	-7.47^b^ ***
*Religion*			6.49**
Protestant	0.37	0.42	
Catholic	0.22	0.24	
Buddhist, or other	0.41	0.34	
*Geographic Region*			12.53**
Remote mountain area	0.12	0.15	
Highlands area	0.37	0.41	
Plains area	0.51	0.44	

*p<.05;

**p<.01;

***p<.001;

^a^Brackets is the standard deviation;

^b^T-test for continuous variable.

Several significant differences of social excluded situations between these two subgroups can be found in the public support for medical, housing, unemployment and financial benefits (p<.001). The happy group is less excluded from welfare provisions compared to their counterparts. Taking some social exclusion situations for example, the percentages of social exclusion for the happy group are: 29%, 51%, 25% and 52%, in accessing medical care, housing, unemployment and financial benefits, respectively. Similar patterns of social exclusion are also found among the unhappy samples. However, the unhappy respondents experienced more difficulties in attempting to receive the abovementioned welfare provisions than their counterparts did. The exclusions from receiving financial assistances and housing benefits programs are the first two problems for the unhappy group in accessing public support, which account for 78% and 65%, respectively. In contrast, there is no statistical difference between the happy and unhappy respondents with respect to senior, child and youth benefits.

The results in Table 2 provide some preliminary evidence that happiness may differ among indigenous adults with different socio-demographic characteristics and those who are excluded from distinct welfare provisions. These conclusions, however, are not necessarily justified, inasmuch as the possible differences in other exogenous factors between groups have not yet been controlled [24]. The following section further employs a binary logistic model to investigate the effects of social exclusion and other exogenous factors on the likelihood of perceiving happiness.

### Determinants of happiness of indigenous peoples

The primary focus of this empirical analysis is on the effect of social exclusion and other exogenous factors on the likelihood of being self-perceived as happy. The estimation results of a binary logistic regression model of happiness are presented in Table 3. In addition to the six domains of social exclusion variables that indicate whether an indigenous person is excluded from accessing different welfare provisions, this study also controls for several variables reflecting the socio-demographic characteristics, physical and mental health status, and regional heterogeneity. All explanatory variables used in the logistic regression models passed the collinearity diagnostics by using the variance inflation factor test (all VIF<10) [25].

**Table 3:** Binary logistic regression on happiness of indigenous peoples (happy vs. unhappy) (N = 2,200).

Explanatory variables	Coef.	S.E.	M.E.	S.E.	P-value
***Social exclusion (ref*. *excluded)***					
*Medical benefits*					
no need	0.154	0.198	0.095	0.107	0.375
not excluded	0.295*	0.154	0.172	0.085	0.044
*Housing benefits*					
no need	0.661***	0.201	0.334	0.105	0.002
not excluded	0.162	0.167	0.084	0.091	0.358
*Child and youth benefits*					
no need	-0.192	0.166	-0.113	0.092	0.220
not excluded	0.008	0.170	0.007	0.093	0.943
*Senior benefits*					
no need	-0.132	0.160	-0.077	0.088	0.378
not excluded	-0.409*	0.194	-0.248	0.107	0.021
*Unemployment benefits*					
no need	0.136	0.161	0.076	0.089	0.396
not excluded	-0.220	0.208	-0.126	0.118	0.284
*Financial assistances*					
no need	0.980***	0.195	0.512	0.098	<.0001
not excluded	0.052	0.248	0.019	0.139	0.892
**Socio-demographic characteristics**					
*Male* (ref. female)	-0.387**	0.139	-0.223	0.077	0.004
*Age under 55 years* (ref. over 55)	-0.363*	0.162	-0.069	0.160	0.033
*Education* (ref. elementary or lower)					
Junior high school	0.213	0.151	0.041	0.152	0.171
Senior high school	0.111	0.184	0.021	0.183	0.544
Vocational school	0.503**	0.162	0.098	0.160	0.001
College or higher	0.310*	0.171	0.062	0.170	0.477
*Health status*					
Physical health status (ref. poor)	0.647***	0.156	0.358	0.082	<.0001
Mental problem (ref. none)	-1.398***	0.266	-0.717	0.126	<.0001
**Other external factors**					
*Household income*	0.095**	0.032	0.047	0.016	0.054
*Religion* (ref. Buddhist or other)					
Protestant	0.351*	0.155	0.213	0.085	0.013
Catholic	0.397*	0.179	0.231	0.099	0.019
*Geographic Region* (ref. plains area)					
Remote mountain area	0.531***	0.152	0.104	0.154	0.001
Highlands area	0.274**	0.114	0.052	0.108	0.010
*Intercept*	1.816***	0.379	1.016	0.197	<.0001

*p<.05;

**p<.01;

***p<.001;

Coef. = coefficient; S.E. = standard error; M.E. = marginal effect;

Likelihood ratio of Chi-Square test = 241.19 (P-value<.001), reject Global Null Hypothesis (β = 0).

As exhibited in Table 3, the estimation results of the happiness equations show that females and older (over 55 years old) indigenous peoples are more likely to be happy compared to their counterparts (p<.005). Moreover, the likelihood of perceiving happiness is higher for those who are wealthier; a 1% point increase in monthly household income increases the likelihood of being happy by 0.047 percentage points (i.e. a marginal effect). The results also show that, compared to those who graduated from elementary school or lower (the reference group), respondents who graduated from vocational school and college or higher education are more likely to be happy, by 9.8% and 6.2%, respectively. This socio-demographic difference in happiness is in accordance with previous findings [7, 23, 26] and suggests that raising occupational competence and income level can enhance subjective well-being, particularly for the poor group. Religion is also a significant factor. Compared with Buddhism or with other religious belief, the indigenous Protestants and Catholics are more likely to be happy by 21.3% and 23.1%, respectively.

Geographic region plays a relevant role in perceiving happiness. Those who reside in remote mountainous and highland areas are more likely to be happy compared to their counterparts, by 10.4% and 5.2%, respectively. This result may reflect the fact that the higher the mountain location, the closer to the traditional territory of indigenous peoples; therefore, they have more chance to continue their aboriginal lifestyles. Perhaps this natural living circumstance and traditional way of life in mountainous areas lead them to be happier. With respect to health status, a positive association between self-reported health and happiness is found. In accordance with the findings in Dockery (2010) [27], the results show that those who are self-reported as physically and mentally healthy are more likely to be happy compared to their counterparts, by 35.8% and 71.7%, respectively.

Finally, perhaps the most interesting finding is in regard to the effects of the social exclusion on happiness. As expected, the results reveal that the likelihood of being happy is higher for those who have received public medical benefits, as well as those without housing problems and financial difficulties, compared to their counterparts who are excluded from the abovementioned welfare provisions. For instance, compared with respondents excluded from receiving medical benefits (i.e. the reference group), those who successfully received medical benefits are 17.2% higher in being happy than their excluded counterparts. Similarly, those who do not need financial assistance are more likely to be happy than those excluded from financial assistance (marginal effect = 51.2%). These results are consistent with the social sustainability hypothesis, in that people are more likely to perceive self-happiness if they are integrated or included in the greater society [2, 28]. However, no statistically significant association is found between happiness and some welfare domains of social exclusion, such as child and youth benefits, and unemployment benefits. Surprising, a negative relationship is evident between whether or not one is excluded from senior benefits and the likelihood of being happy. This undesired outcome may reflect the fact that the existing public senior benefits do not actually meet the practical needs of caring for the indigenous elders. Another possible explanation is that perhaps the senior respondents who are excluded from receiving welfare have found their own way to take care of family elders. As a result, those who have received public senior benefits are more likely to be unhappy than their excluded counterparts, by 24.8%.

## Conclusions

Increasing happiness and reducing social exclusion are strongly recommended for realizing the Sustainable Development Goals of the UN [1, 2, 29]. Although extensive literature has examined the factors that are associated with indigenous peoples’ development, relatively little is known about their subjective wellbeing [20, 30–32]. To narrow this knowledge gap, this paper employed the perspective of social sustainability to examine the effects of social exclusion, as well as personal and external determinants on the happiness of indigenous peoples in Taiwan. Using nationwide TIPS data in Taiwan, the empirical results show that socio-demographic characteristics, health-related factors and geographic region are significantly associated with indigenous people’s happiness.

The disengagement of the indigenous peoples with respect to the accessibility of social welfare provisions is a crucial element in regard to social exclusion and happiness. Further empirical results suggest that health status and medical social exclusion are the most pronounced determinants of happiness. In addition, those who have no need for housing benefits and financial assistances are more likely to be happy, compared to their socially excluded counterparts. Surprisingly, an unexpected result finds that successfully receiving senior benefits has an adverse effect on happiness, compared to their excluded counterparts. Perhaps, this finding reflects the fact that the indigenous tribal solidarity and social network system may serve more active functions than public welfare agents in caring for the elders. Those who have a weak private support system for caring for tribal elders, and thus have to depend on public senior benefits, will perceive being less happy.

This study provides valuable insights into reshaping the indigenous development policy in Taiwan. Several policy implications for social sustainability of indigenous peoples can be inferred from these findings. First, since current social welfare provisions can just keep indigenous peoples in minimal living conditions, rather than eliminating socio-economic and environmental disadvantages, the indigenous development policy might pay more attention to enhancing subjective wellbeing and strengthening social inclusion in the future. Moreover, since mountain indigenes tend to confront challenges in accessing medical healthcare resources associated with remoteness, as well as a lack of social agencies, providing a mobile clinical tour, free pick-up service for medical care, on-site health counseling, or home care service to mountain indigenes is an alternative which can remove institutional and geographic barriers of medical welfare provisions. Additionally, the traditional tribal lifestyle is both an adaptation and reaction of the indigenous peoples to their daily life with environment constraints. It is crucial for the indigenous social policy to maintain or strengthen the traditional way of life. For instance, the family and community are often posited as an alternative to the delivery of social welfare provisions, as well as the major sources of pursuing happiness. Hence, the authorities may devote more welfare resources to assist indigenous communities to develop their own social safety net, instead of relying merely on individual-oriented welfare provisions. Such family- and community-based supporting measures will help to reduce the social exclusion related to seniors, child and youth care, as well as employment in indigenous tribal areas.

Although some interesting findings are revealed in this study, some study limitations pertain. Firstly, the Taiwanese Indigenous Peoples Survey only investigate the needs and reception status of specific social welfare packages, rather than individual items of social benefits received by indigenous peoples. It will be relevant to further investigate how different social welfare measures, such as in-kind benefits or cash payment, may determine the likelihood of perceiving self-happiness. Further data limitation is that the TIPS did not provide social network, lifestyle, or psychological factors of the indigenous peoples. Such social life conditions of the indigenous peoples are essential to show the robustness of this study’s findings. This issue could be better addressed if detailed information of separate welfare items and social lifestyle of respondents were available.
